# Evaluating the Impact of Oleocanthal and Oleacein on Skin Aging: Results of a Randomized Clinical Study

**DOI:** 10.3390/medicina60060947

**Published:** 2024-06-06

**Authors:** Steven P. Nisticò, M. E. Greco, S. Amato, L. Bennardo, E. Zappia, E. Pignataro, G. Pellacani

**Affiliations:** 1 Dermatology Unit, Department of Clinical Internal Anesthesiologic Cardiovascular Sciences, Sapienza University of Rome, 00185 Rome, Italymariaelisabetta.greco@uniroma1.it (M.E.G.);; 2 Department of Health Sciences, Magna Grecia University, 88100 Catanzaro, Italy; 3 Department of Experimental Medicine, Sapienza University of Rome, 00185 Rome, Italy; elia.pignataro@uniroma1.it

**Keywords:** skin aging, wrinkle reduction, extra virgin olive oil, oleocanthal, oleacein polyphenols, VISIA^®^ skin analysis system, dermatological research, anti-aging skincare, clinical trial

## Abstract

The prevalence of skin aging and the request for effective treatments have driven dermatological research towards natural solutions. This study investigates the anti-aging efficacy of two bioactive natural polyphenols, Oleocanthal and Oleacein, in a skincare formulation. A single-blind, randomized clinical trial involved 70 participants, using a comprehensive exclusion criterion to ensure participant safety and study integrity. Participants applied the Oleocanthal and Oleacein 1% serum formulation twice daily for 30 days. The efficacy was objectively assessed using the VISIA^®^ Skin Analysis System at baseline, after 15 days, and after 30 days. Results indicated significant wrinkle reduction in most groups. For women aged 45–79 years, the mean change was −33.91% (95% CI: −46.75% to −21.07%). For men aged 20–44 years, it was −51.93% (95% CI: −76.54% to −27.33%), and for men aged 45–79 years, it was −46.56% (95% CI: −58.32% to −34.81%). For women aged 20–44 years, the change was −25.68% (95% CI: −63.91% to 12.54%), not statistically significant. These findings highlight the potential of EVOO-derived polyphenols in anti-aging skincare, particularly for older adults. This research paves the way for further exploration into natural compounds in dermatology, particularly for aging skin management.

## 1. Introduction

The pursuit of natural, effective anti-aging treatments is a growing area of interest, particularly in the field of dermatology.

Wrinkles, a key indicator of skin aging, arise from both intrinsic factors, such as changes in dermal and epidermal structures, and extrinsic factors like UV exposure and lifestyle choices. These factors contribute to decreased fibroblast activity and reduced collagen production, accelerating wrinkle formation.

Extra Virgin Olive Oil (EVOO) and its extracts play a central role in counteracting aging sign—It is recognized not only for its role in the Mediterranean diet but also for its broader health benefits. These benefits have been shown in many studies and include cardiovascular protection, anti-inflammatory effects, and metabolic health benefits [1,2,3,4,5]. Actuality, olive oil consumption can lessen inflammation, endothelial dysfunction, lipid and DNA oxidation, lipid profile, insulin resistance, and blood pressure in hypertensive people. These benefits guard against metabolic abnormalities as well as cardiovascular disease [6].

EVOO is particularly rich in triglyceride andit possesses other limited bioactive components such as sterols, vitamins, escualene, polyphenols, and others. It is rich in polyphenols and secoiridoids like hydroxytyrosol, tyrosol, and oleocanthal and oleacein, which exhibit antibacterial and anti-inflammatory properties [7,8,9].

Additionally, it has been discovered that fatty acids and monoglycerides exhibit a wide range of microbicidal activity against yeasts and bacteria [10]. Additionally, it has been shown that the α,β-unsaturated aldehydes found in olives and olive oil flavor have a discernible effect on pathogens that affect the human respiratory and intestinal tracts [11].

Concerns regarding phenolic compounds from plants and foods that may alter gut microbiota by favoring the growth of lactobacilli and bifidobacteria while suppressing the growth of pathogenic bacteria like clostridia have been raised in relation to human health [12,13].

These compounds have also showed potential in improving dermatological signs related to aging and in particular [14] diminishing wrinkle appearance, positioning EVOO as a key focus in anti-aging research.

Oleocanthal and oleacein, compounds derived from EVOO, have shown promise in skin health enhancement, particularly in treating non-melanoma skin cancers and facilitating wound healing [15,16]. Their effectiveness aligns with broader research on the skin health benefits of polyphenols, including their ability to penetrate epidermal barriers and interact with cellular receptors.

This study focuses for the first time on a detailed evaluation of a dermatological serum containing Oleocanthal and Oleacein, EVOO-derived polyphenols, focusing on its anti-wrinkle efficacy through multispectral analysis. Our aim is to provide a comprehensive, scientifically robust analysis of wrinkle reduction across various demographics, contributing to the field of natural anti-aging solutions.

EVOO, with its rich composition of beneficial compounds, is proving to be a versatile agent in skin health, extending beyond dietary benefits to topical applications. The polyphenols Oleocanthal and Oleacein, two polyphenols derived from EVOO have been incorporated into skincare products to gain more natural, sustainable approaches in anti-aging treatments. Moreover, traditional antiaging compounds are being enhanced by natural ingredients and innovative technologies, paving the way for more effective, holistic treatment strategies. This convergence of natural ingredients and advanced technology is not only redefining treatment protocols but also aligning with a growing consumer preference for sustainable, health-conscious solutions in skincare and medical treatments.

In this open randomized clinical study, we investigate the antiaging efficacy of Oleocanthal and Oleacein in an innovative skincare formulation.

While this study provides valuable insights into the efficacy of Oleocanthal and Oleacein, it is important to note the limitations associated with the functional status of patients, including issues related to sensitization. Participants’ skin sensitivity and reactions to the serum formulation can vary widely, potentially affecting the outcomes. Sensitization to active ingredients is a critical factor that can influence the effectiveness and safety of dermatological treatments. Therefore, careful monitoring and consideration of individual skin reactions are essential to ensure accurate and reliable results.

## 2. Materials and Methods

### 2.1. Study Design and Setting

This study was a prospective, controlled, randomized, single-blind clinical trial conducted at the Dermatology Units of Magna Graecia University in Catanzaro and Sapienza University in Rome, Italy. The study was a prospective, controlled, randomized single-blind clinical trial conducted at the Dermatology Units of Magna Graecia University in Catanzaro and Sapienza University in Rome, Italy. The study was carried out from May to June 2023 targeting individuals with mild to moderate signs of aging. The participants were divided into subgroups based on age and skin morphotype: women (31 aged 20–44 years, 24 aged 45–79 years) and men (8 aged 20–44 years, 7 aged 45–79 years). Each subgroup was evenly distributed between ‘thin skin’ and ‘thick skin’ morphotypes.

### 2.2. Participants

Seventy healthy immunocompetent males and females aged between 18 and 75 years were recruited. The sample is divided into women, with 31 participants aged 20–44 years and 24 participants aged 45–79 years, evenly distributed across two morphotype groups: ‘thin skin’ and ‘thick skin’. The sample also includes men, with 8 participants aged 20–44 years and 7 participants aged 45–79 years, similarly distributed across the two morphotype groups. The classification of skin morphotype was determined using high-frequency ultrasound, which measured the epidermal and dermal thickness of the facial skin. Mean values of epidermal thickness were approximately 0.18 mm, while mean values of dermal thickness were around 0.98 mm. Participants with dermal thickness values significantly above or below these means were classified as having ‘thick skin’ or ‘thin skin,’ respectively. The study enforced strict exclusion criteria to ensure the reliability and safety of the results. Excluded were individuals with extremely mild or severe skin aging, a history of skin cancer on the face or scalp, systemic tumors, dermatological conditions.

### 2.3. Informed Consent and Data Collection

All participants provided informed consent at their baseline visit. Detailed data, including age, Fitzpatrick skin phototype, medical history, chronic medication use, smoking and alcohol habits, weight, and height, were meticulously recorded. The study adhered to current legislation for observational studies, Good Clinical Practice (GCP) guidelines, and the ethical principles of the Declaration of Helsinki.

### 2.4. Treatment Protocol

Participants applied a biphasic serum containing Oleocanthal and Oleacein EVOO-derived polyphenols, (PureXerum^®^, Active-Italia, Rome, Italy) twice daily for 30 days. The formula contained: Propandiol, Prunus amygdalus duclis oil and Betaine as vehicles and Tocopheryl acetate, Oleacein and Oleocanthal (1%) as active principles.

### 2.5. Evaluation Schedule

Patient evaluations occurred at three key intervals: baseline (t0), after 15 days (t1), and after 30 days (t2) of product usage. The first follow-up visit was conducted after 15 days, with 50 participants completing the third visit.

### 2.6. Objective Assessment

Skin aging improvement was objectively evaluated using the VISIA^®^ Skin Analysis System (Canfield Scientific, Parsippany, NJ, USA). This system provided a comprehensive and objective measurement of the treatment’s impact on skin, assessing both the total area of wrinkles and their intensity. The VISIA^®^ system is validated through multiple studies and offers a detailed visualization of skin roughness and treatment progress.

### 2.7. Statistical Analysis

For this study, we used specialized statistical software SPSS (Statistical Package for the Social Sciences) that was fully compatible with the VISIA® Skin Analysis System to analyze the data. This software enabled the precise quantification of various skin parameters with a particular focus on wrinkle metrics. To determine statistical significance, 95% Confidence Intervals (CIs) were calculated for key metrics, including mean wrinkle count and percentage change from baseline. A 95% CI that did not include zero was considered indicative of statistically significant changes.

Data were presented in two formats: absolute scores and percentage changes from the baseline, allowing for a nuanced comparison of wrinkle progression throughout the treatment period. We conducted an inclusive analysis of both superficial and deep wrinkles to provide a comprehensive assessment of Oleocanthal and Oleacein serum efficacy. Recognizing the importance of gender-specific responses, separate analyses for male and female participants were also carried out.

To facilitate the interpretation of our findings, graphical data such as forest plots were used. This visual representation helped in highlighting key trends and patterns. The study collected longitudinal data at three time points—baseline, 15 days, and 30 days post-treatment initiation—enabling an evaluation of the treatment’s efficacy over time.

## 3. Results

The data from this study offer robust evidence supporting the efficacy of 1% Oleocanthal and Oleacein serum in reducing wrinkles. Employing the VISIA^®^ imaging system for precise measurements, we observed a significant decrease in wrinkle count across all participants (Figure 2, Figure 3, Figure 4 and Figure 5). Notably, there was an average percentage reduction of 23.1% in wrinkle count at the end of the 30-day period compared to baseline measurements.

**Figure 1:**
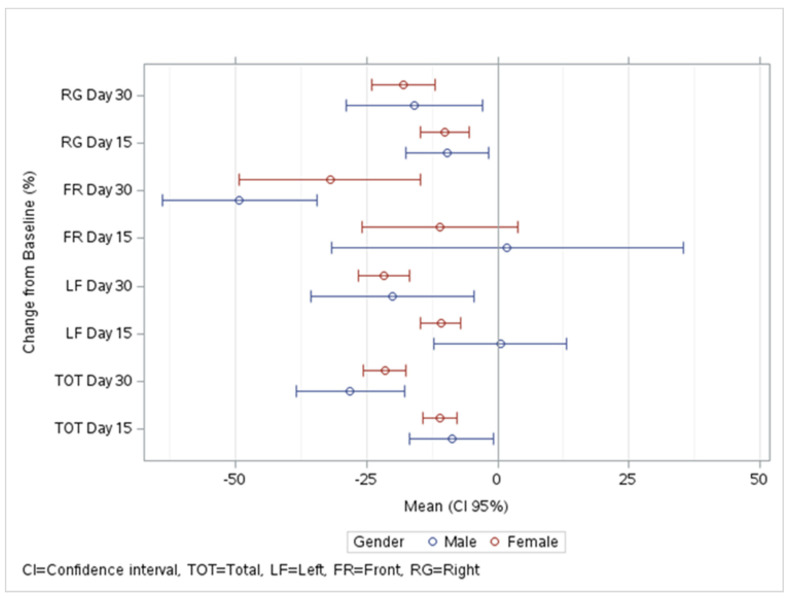
Change from Baseline in Wrinkle Count by Region and Gender.

A consistent trend of decreasing wrinkle counts was evident at each of the three evaluation intervals: baseline, 15 days, and 30 days (Figure 1). Specifically, for men, the median wrinkle count at the start was 83,232, which reduced to 70,568 at day 15, and further to 56,735 by day 30. Similarly, for women, the median count decreased from 76,369 at baseline to 70,441 at day 15, and then to 61,491 at day 30. This trend demonstrates not only the effectiveness of the treatment in wrinkle reduction but also its sustained impact over the course of the study.

**Figure 2:**
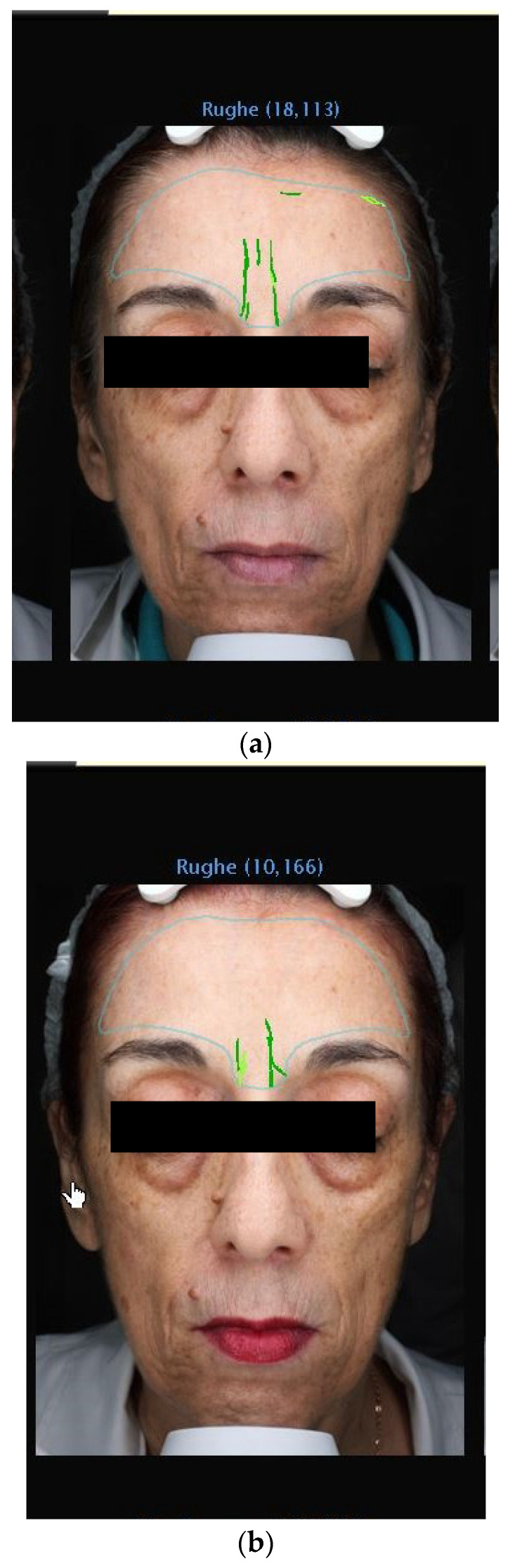
(**a**) VISIA Image Analysis of Female Patient: T1—Detailed Mapping of Facial Wrinkles. (**b**) VISIA Image Analysis of Female Patient: T3—Detailed Mapping of Facial Wrinkles.

**Figure 3:**
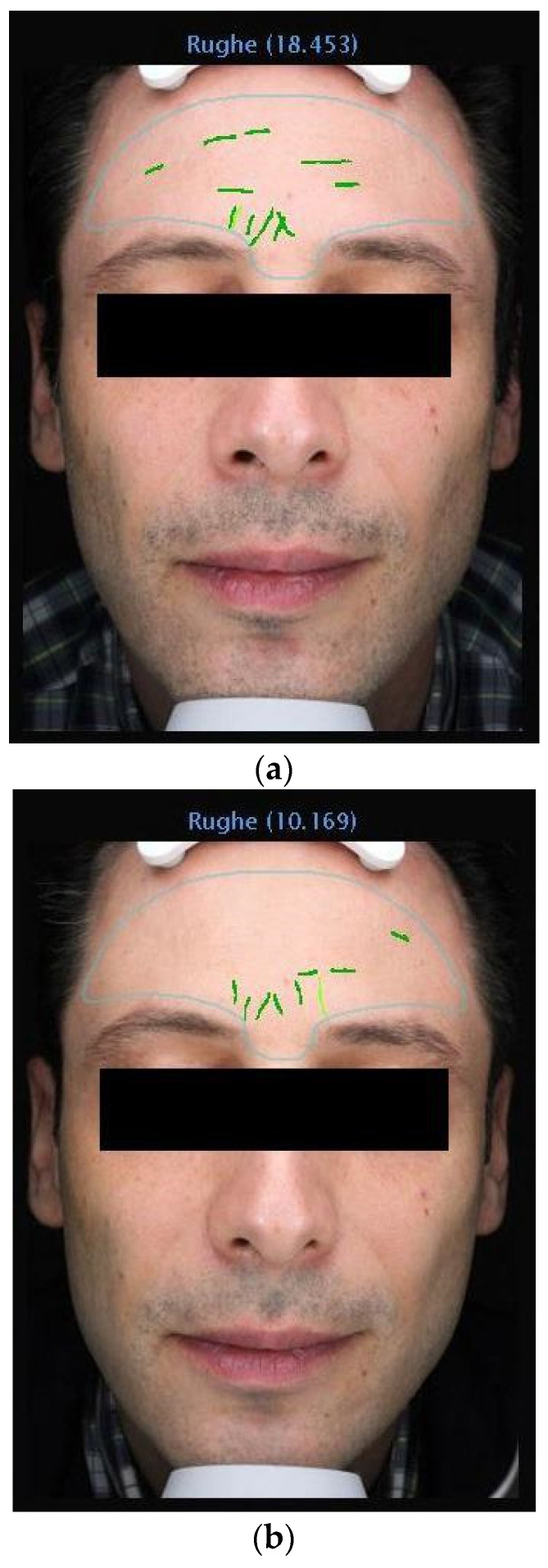
(**a**) VISIA Image Analysis of Male Patient: T1—Detailed Mapping of Facial Wrinkles. (**b**) VISIA Image Analysis of Male Patient: T3—Detailed Mapping of Facial Wrinkles.

**Figure 4:**
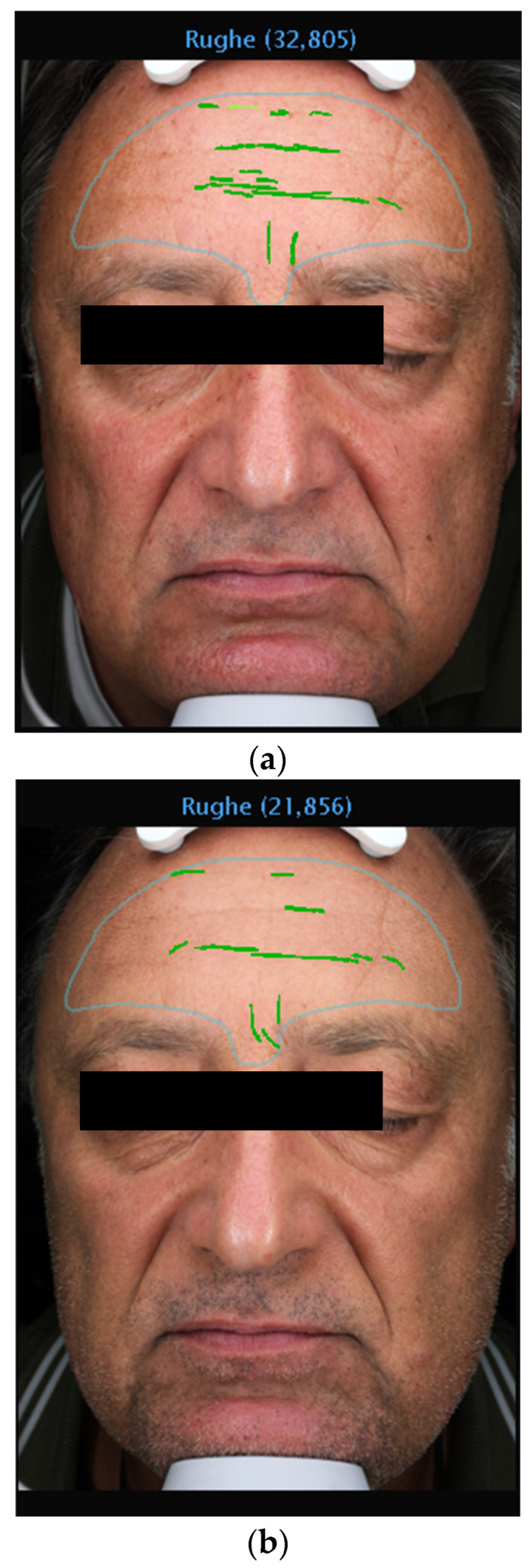
(**a**) VISIA Image Analysis of Male Patient: T1—Detailed Mapping of Facial Wrinkles. (**b**) VISIA Image Analysis of Male Patient: T3—Detailed Mapping of Facial Wrinkles.

**Figure 5:**
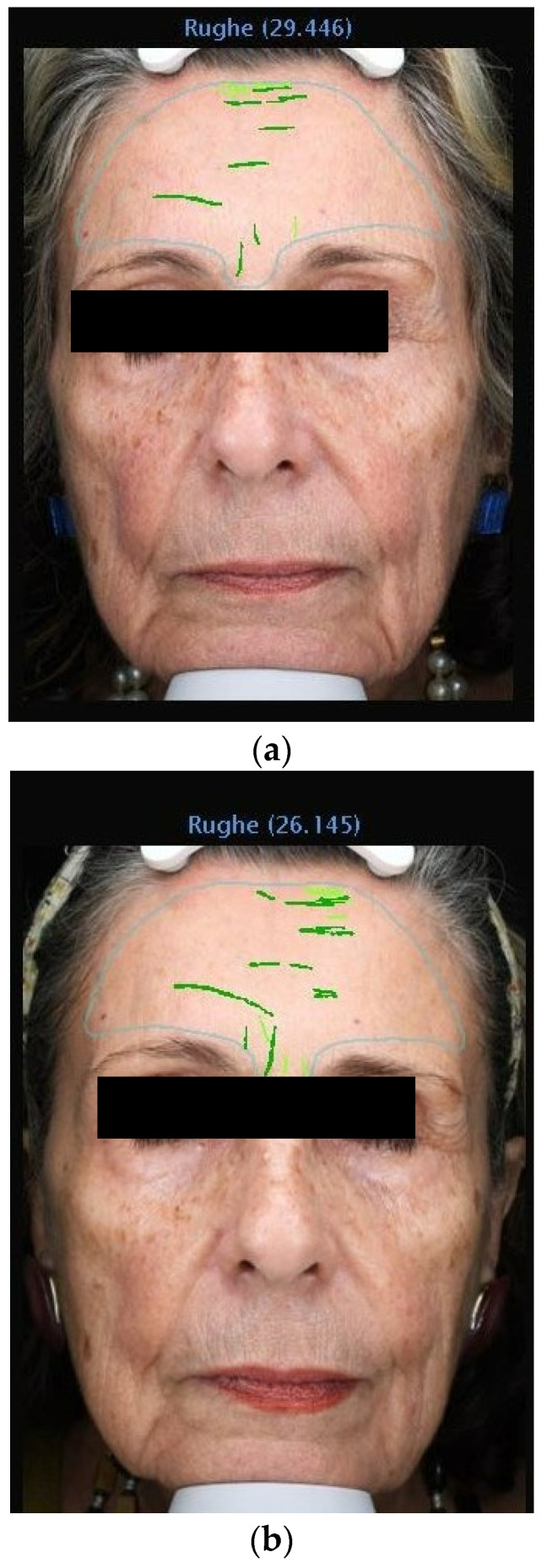
(**a**) VISIA Image Analysis of Male Patient: T1—Detailed Mapping of Facial Wrinkles. (**b**) VISIA Image Analysis of Male Patient: T3—Detailed Mapping of Facial Wrinkles.

### 3.1. Generalizability of Findings

The findings were further substantiated by the Interquartile Range (IQR) scaling, which revealed that the positive outcomes of the treatment were consistent across different demographic segments. This indicates that Oleocanthal and Oleacein’s effectiveness in wrinkle reduction is not confined to a specific subgroup but is broadly applicable, thereby enhancing the generalizability of the results. The significant reduction in wrinkle counts in both male and female participants underscore the serum containing Oleocanthal and Oleacein’s potential as an effective anti-wrinkle treatment.

### 3.2. Number of Wrinkles: Absolute Scores

Detailed data on the number of wrinkles will be presented here, captured at different time intervals: baseline, day 15, and day 30. The data will be recorded as “absolute scores”, providing a precise quantitative snapshot of the participants’ skin conditions. Both surface wrinkles and deep wrinkles will be included, as measured by the VISIA^®^ camera system. The following Table 1 presents the absolute scores for wrinkle count at baseline, day 15, and day 30.

### 3.3. Number of Wrinkles: Percent Change from Baseline

This section will delve into the percentage change in the number of wrinkles from the baseline. It will provide a clear picture of the treatment’s efficacy over time. The data will be broken down by gender to highlight any differences in treatment response between men and women (Table 2).

### 3.4. Gender-Specific Results

**Figure 6:**
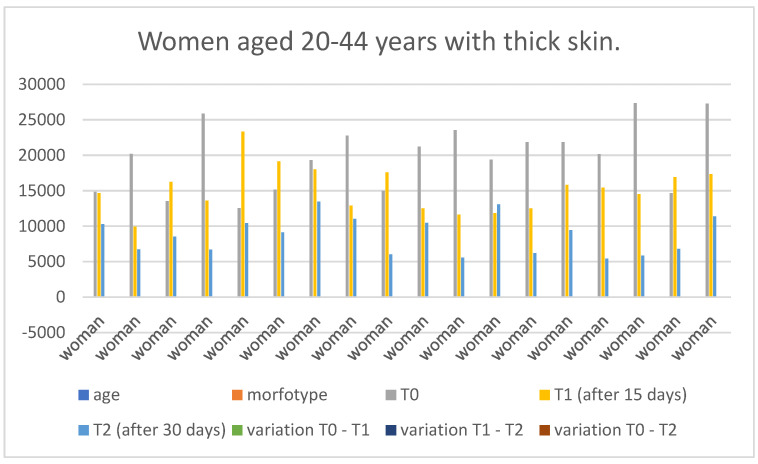
Graph related to the data exhibited in Table 3. This graph shows visually the results on the sample of women aged 20–44 years with thick skin at time 0, 1, and 2.

**Figure 7:**
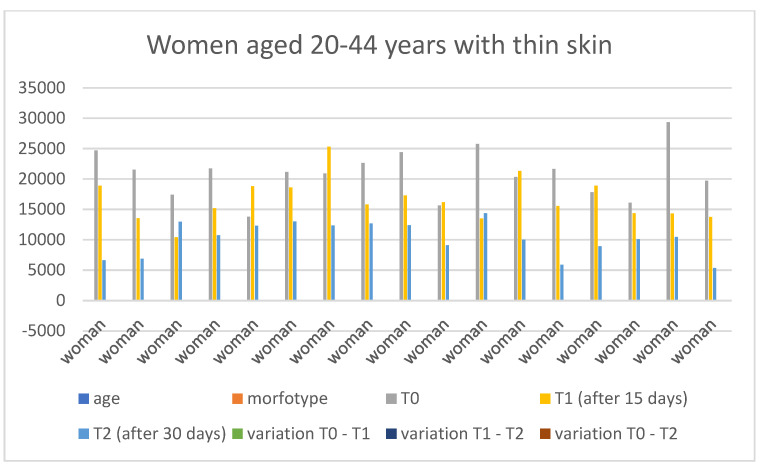
Graph related to the data exhibited in Table 4. This graph shows visually the results on the sample of women aged 20–44 years with thin skin at time 0, 1, and 2.

**Figure 8:**
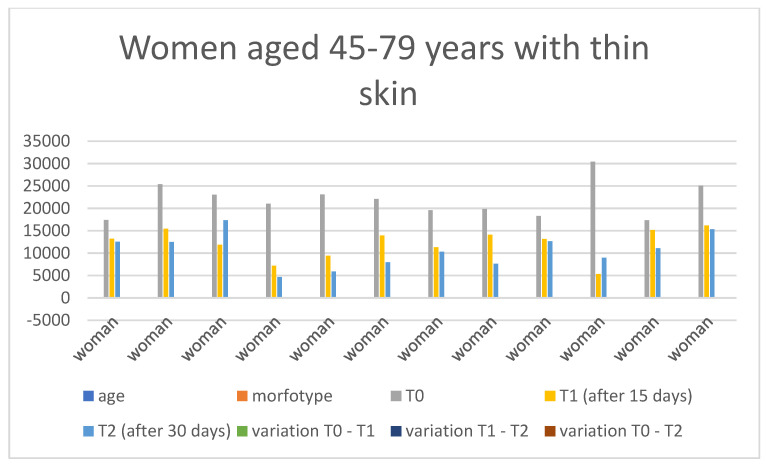
Graph related to the data exhibited in Table 5. This graph shows visually the results on the sample of women aged 45–79 years with thin skin at time 0, 1, and 2.

**Figure 9:**
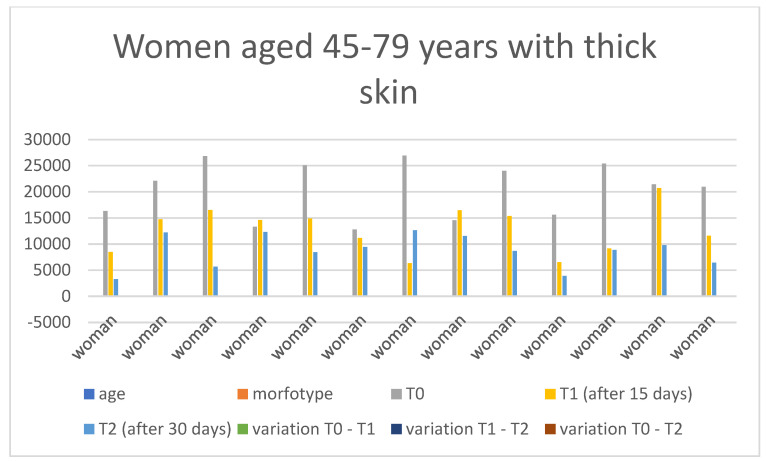
Graph related to the data exhibited in Table 6. This graph shows visually the results on the sample of women aged 45–79 years with thick skin at time 0, 1, and 2.

**Figure 10:**
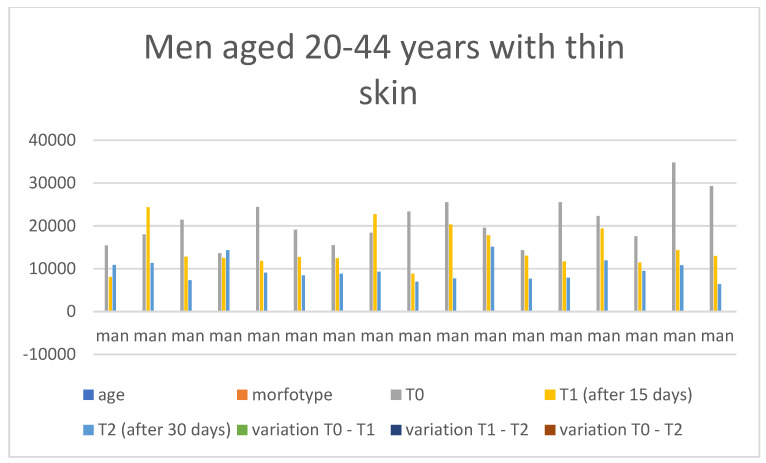
Graph related to the data exhibited in Table 7. This graph shows visually the results on the sample of men aged 20–44 years with thin skin at time 0, 1, and 2.

**Figure 11:**
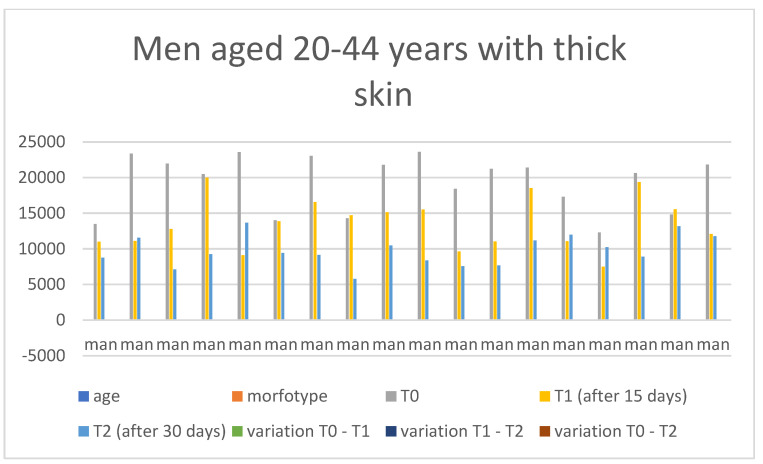
Graph related to the data exhibited in Table 8. This graph shows visually the results on the sample of men aged 20–44 years with thick skin at time 0, 1, and 2.

**Figure 12:**
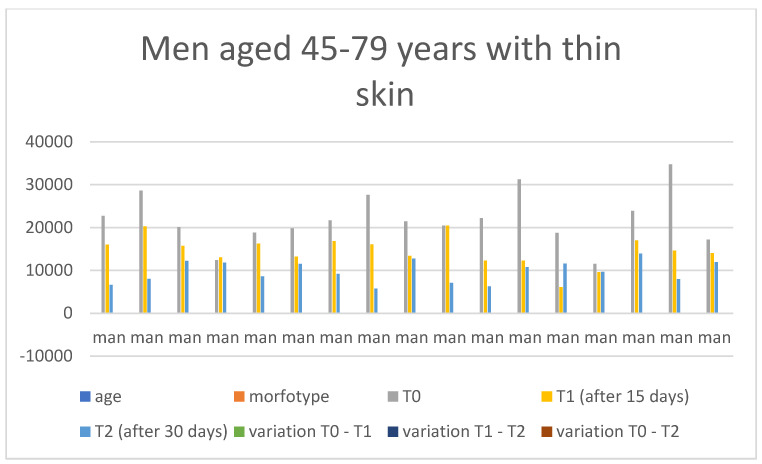
Graph related to the data exhibited in Table 9. This graph shows visually the results on the sample of men aged 45–79 years with thin skin at time 0, 1, and 2.

**Figure 13:**
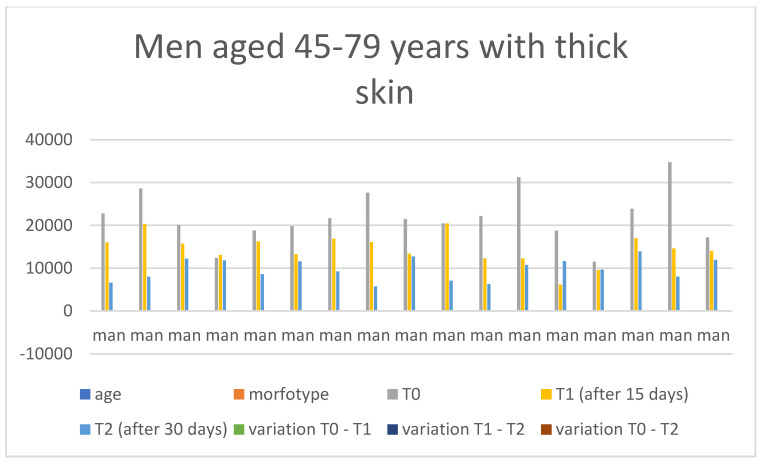
Graph related to the data exhibited in Table 10. This graph shows visually the results on the sample of men aged 45–79 years with thick skin at time 0, 1, and 2.

Below are the tables with the results, organized as follows: Women aged 20–44 years with thick skin (Table 3), Women aged 20–44 years with thin skin (Table 4), Women aged 45–79 years with thin skin (Table 5), Women aged 45–79 years with thick skin (Table 6), Men aged 20–44 years with thin skin (Table 7), Men aged 20–44 years with thick skin (Table 8), Men aged 45–79 years with thin skin (Table 9), and Men aged 45–79 years with thick skin (Table 10). Each table includes information on age, morphotype according to skin thickness, and wrinkle measurements at time points T0, T1, and T2. Additionally, the tables report the variations in values for the intervals: variation T0–T1, variation T1–T2, and variation T0–T2. Graphical representations have been included to enhance data visualization (Figure 6, Figure 7, Figure 8, Figure 9, Figure 10, Figure 11, Figure 12 and Figure 13). These figures, presented as multiple bar charts, illustrate the changes in wrinkle measurements over time and the variations between these points, providing a clearer depiction of the results.

#### 3.4.1. Men’s Data Analysis

The data shows a clear reduction in the number of wrinkles in male patients over 30 days of treatment. While there is considerable variability among individuals, the median and mean values demonstrate consistent decrease in wrinkle count from baseline through 15 days to 30 days across all measured facial areas. After 30 days, the average reduction in wrinkle count for men aged 20–44 years was −51.93% (95% CI: −76.54% to −27.33%), and for men aged 45–79 years, it was −46.56% (95% CI: −58.32% to −34.81%), indicating that the treatment is generally effective in reducing wrinkle count in male patients.

#### 3.4.2. Women’s Data Analysis

The data analysis for women reveals a reduction in average wrinkle counts after 15 and 30 days of treatment. Baseline median wrinkle count was 76,369, which decreased to 70,441 after 15 days, and further to 61,491 after 30 days. For women aged 45–79 years, the mean percentage change in wrinkle count was −33.91% (95% CI: −46.75% to −21.07%), indicating a statistically significant reduction in wrinkles. However, for women aged 20–44 years, the change was −25.68% (95% CI: −63.91% to 12.54%), not statistically significant, suggesting a need for further research to understand the variability in this subgroup.

Our study demonstrates that Oleocanthal and Oleacein significantly reduce wrinkle count in both men and women, particularly in those aged 45–79 years. The data collected at three distinct time intervals reveals a consistent trend of wrinkle reduction. However, the overall findings support the use of EVOO-derived polyphenols in anti-aging skincare formulations.

Initial Counts: At the onset of the treatment, men had a median wrinkle count of 83,232, and women had 76,369. Mid-Treatment: After 15 days, the median wrinkle count decreased to 70,568 for men and 70,441 for women, indicating a positive treatment effect. End of Treatment: This trend continued through day 30, with the median wrinkle count further reducing to 56,735 for men and 61,491 for women. The Interquartile Range (IQR) scaling suggests greater uniformity in treatment outcomes over time. This indicates that the treatment’s efficacy is not limited to a specific subgroup but is broadly applicable, enhancing its generalizability.

## 4. Discussion

Extra Virgin Olive Oil (EVOO) is rich in biologically active compounds such as oleocanthal and oleacein [17,18]. These compounds are known for their anti-inflammatory properties and their ability to inhibit COX-1 and COX-2 enzymes, mirroring the action of synthetic drugs like ibuprofen but without the associated side effects [19]. Our findings align with recent studies like those by Melguizo-Rodríguez et al. [20] and Donato-Trancoso et al. [21], which explore the biological effects of olive tree derivatives on the skin.

As we have already mentioned, olive oil contains triglyceride, monoglyceride, and diglyceride fatty acids make up the majority of its composition. More than 230 different compounds make up EVOO’s minor compounds, which make up 2% of its total weight. These include hydrocarbons, sterols, aliphatic alcohols, tocopherols, pigments, volatile compounds, and phenolic compounds, which primarily consist of flavonoids, lignans, phenolic acids, phenolic alcohols, and secoiridoids and are related to the significant antioxidant potential of phenolic compounds [22,23].

Furthermore, the complex interplay of genetic and environmental factors—like UV radiation and pollution—in skin aging suggests that these compounds can positively impact skin aging, slowing down the process and promoting healthier, younger-looking skin [24].

Our results are also consistent with existing scientific literature on the benefits of polyphenols for skin health and overall health. These findings offer strong proof that olive oil and its phenolic component can be utilized as an alternative dietary therapy to prevent and treat oxidative and inflammatory immunological processes [25].

Polyphenols are known to enhance the skin’s endogenous antioxidant system and can cross epidermal barriers to interact with cellular receptors, thereby preventing various skin diseases. They have been observed to improve the structural organization of the dermis, crucial for its barrier function, and to increase dermis thickness and hydration levels, essential for maintaining healthy, well-functioning skin [26].

The anti-inflammatory properties of EVOO’s polyphenols are significant in preventing and treating immune-mediated inflammatory diseases [27], supporting our findings on the skin health benefits of EVOO. EVOO’s derivatives have a profound impact on immune-inflammatory responses, highlighting its potential in clinical applications related to skin health [28]. The protective effects of EVOO on immune-mediated inflammatory responses align with our observations on skin aging [21].

Moreover, recent studies have highlighted the role of EVOO in enhancing skin repair mechanisms [29,30,31]. The topical application of EVOO has been shown to accelerate wound healing processes, attributing to the stimulation of collagen synthesis and angiogenesis. This finding is particularly relevant considering the increasing prevalence of skin conditions requiring regenerative treatments.

The application of EVOO in cosmetic formulations has gained attention due to its ability to improve skin elasticity and texture. Studies suggest that regular use of EVOO-based products can lead to a significant reduction in the appearance of fine lines and wrinkles, offering a natural alternative to synthetic anti-aging products.

This research, carried out at the University La Sapienza of Rome, importantly contributes to our understanding of anti-aging skincare. Our study focused on the effects of Oleocanthal and Oleacein, key ingredients in a polyphenol rich EVOO skincare formulation. In a carefully designed single-blind, randomized trial involving 70 participants, we noted a significant reduction in wrinkles. For women aged 45–79 years, the mean change was −33.91% (95% CI: −46.75% to −21.07%). For men aged 20–44 years, it was −51.93% (95% CI: −76.54% to −27.33%), and for men aged 45–79 years, it was −46.56% (95% CI: −58.32% to −34.81%). For women aged 20–44 years, the change was −25.68% (95% CI: −63.91% to 12.54%), not statistically significant. These significant findings not only affirm the objectives of our study but also emphasize the value of natural compounds in dermatological treatments, particularly for aging skin management. The uniformity of these results across various genders and facial regions highlights the broad applicability of EVOO-derived polyphenols in anti-aging skincare. This aligns with, and contributes to, the growing evidence supporting the effectiveness of nature-based solutions in enhancing skin health.

## 5. Conclusions

This research rigorously evaluated the efficacy of Oleocanthal and Oleacein, an Active-Italia skincare product, in reducing wrinkles. Utilizing the VISIA^®^ imaging system, we observed significant decreases in wrinkle counts across most demographic groups over a 30-day period. The results, coupled with the product’s safety and tolerability profile, establish Oleocanthal and Oleacein as a viable option for wrinkle reduction and overall skin health enhancement.

Future research should aim to understand the variability in response among younger women and explore the long-term effects of Oleocanthal and Oleacein. Additionally, examining the specific mechanisms by which oleocanthal and oleacein contribute to wrinkle reduction could offer valuable insights into new skincare innovations. The results, coupled with the product’s safety and tolerability profile, establish Oleocanthal and Oleacein as a viable option for wrinkle reduction and overall skin health enhancement.

The study adds to the evidence supporting natural compounds in skincare, particularly underscoring the benefits of polyphenols. These compounds effectively penetrate epidermal barriers and interact at the cellular level, enhancing skin health. Their presence in Oleocanthal and Oleacein is a crucial factor in its efficacy.

Considering the promising outcomes of this study, it is imperative to explore the long-term effects of Oleocanthal and Oleacein. Future research should aim to understand its continued impact on skin health over extended periods and examine its synergistic effects when used in combination with other skincare treatments. Additionally, delving into the specific mechanisms by which oleocanthal and oleacein contribute to wrinkle reduction could offer valuable insights into new skincare innovations.

Oleocanthal and Oleacein not only demonstrates efficacy in wrinkle reduction but also aligns with the broader scientific understanding of skin health. Its successful application in this study suggests its potential for both cosmetic and therapeutic use. The integration of natural, scientifically proven ingredients like oleocanthal and oleacein into skincare products represents a significant step forward in the quest for more effective, natural anti-aging solutions. This approach not only meets the growing consumer demand for products with natural ingredients but also opens new avenues for future dermatological research and product development. In conclusion, Oleocanthal and Oleacein show how well traditional skincare practices and modern science can work together, offering a compelling example of the advancements achievable in the realm of natural skincare solutions.

In conclusion, while this study provides valuable insights into the efficacy of Oleocanthal and Oleacein serum, its limitations include a short 30-day duration, potential biases due to its single-blind design, and a lack of long-term follow-up and placebo control, which may affect the generalizability and definitive interpretation of the results.

## Figures and Tables

**Table 1:** Absolute scores for wrinkle count at baseline, day 15, and day 30.

	Position	N	Mean	95% CI Low	95% CI High	STD	Minimum	Q1	Median	Q3	Maximum
Baseline	Left side	70	32,974.3	31,084	34,865	7929.0	13,553	27518	34,462	38,178	47,702
	Front	70	14,129.4	11,338	16,921	11,709	1.0	5920.0	10,409	20,238	49,777
	Right side	70	32,832.4	30,915	34,750	8041.6	11,389	28,561	33,849	38,158	50,747
	Full face	70	79,936.2	75,140	84,732	20,115	26,561	67,758	82,160	93,376	125,680
Day 15	Left side	70	29,662.3	27,934	31,391	7248.4	11,757	25,911	29,816	33,310	50,146
	Front	70	12,310.7	9679.6	14,942	11,035	1.0	3930.0	9274.5	17,943	46,782
	Right side	70	29,031.1	27,352	30,710	7041.9	10,540	24,918	30,123	33,132	45,950
	Full face	70	71,004.1	66,482	75,526	18,965	22,298	58,540	71,068	82,350	125,950
Day 30	Left side	50	24,663.4	22,458	26,869	7759.9	6970.0	20,427	24,810	30,024	42,764
	Front	50	8743.2	6530.2	10,956	7786.8	1.0	2947.0	6289.0	12,464	30,841
	Right side	50	25,871.5	23,780	27,963	7359.0	4630.0	20,779	27,487	30,919	39,340
	Full face	50	59,278.1	54,068	64,488	18,334	19,773	49,387	60,577	71,719	111,153

**Table 2:** Number of Wrinkles–Percent Change from Baseline.

	Position	N	Mean	95% CI Low	95% CI Max	STD	Minimum	Q1	Median	Q3	Maximum
Day 15	Left side	70	−8.5	−12.5	−4.4	17.0	−38.5	−19.9	−11.5	0.1	41.8
	Front	70	−8.3	−21.7	5.0	55.9	−99.9	−50.3	−11.1	17.6	145.8
	Right side	70	−10.1	−14.0	−6.2	16.5	−44.9	−21.8	−10.8	−2.0	37.7
	Full face	70	−10.5	−13.5	−7.5	12.7	−36.1	−18.9	−11.1	−3.0	27.1
Day 30	Left side	50	−21.3	−26.2	−16.4	17.4	−59.3	−30.2	−21.5	−6.4	21.9
	Front	50	−36.1	−49.6	−22.6	47.6	−100.0	−53.3	−38.5	−18.8	223.9
	Right side	50	−17.5	−22.8	−12.1	18.9	−67.7	−28.1	−15.4	−7.2	29.9
	Full face	50	−23.1	−27.0	−19.3	13.5	−49.9	−33.7	−23.3	−12.6	11.5

N: This represents the total number of subjects or samples included in the analysis. A larger sample size can provide more accurate estimates. Mean: This is the average value of the sample. In the context of this study, it represents the average percentage change in the number of wrinkles. CI (Confidence Interval): This is a range within which the true mean value (of the population) is expected to occur 95% of the time if the study were repeated many times. The lower and upper confidence interval provides a range of values that are statistically probable given the measures taken. If the confidence interval for the mean does not include zero, it suggests that there is a significant difference.

**Table 3:** Women aged 20–44 years with thick skin.

Gender	Age	Morfotype	T0	T1 (after 15 Days)	T2 (after 30 Days)	Variation T0–T1	Variation T1–T2	Variation T0–T2
woman	28	thick skin	24,700.62	18,879.65	6606.035	−0.23843	−0.31959	−0.42748
woman	36	thick skin	21,513.73	13,520.29	6869.383	−0.25112	−0.32096	−0.52694
woman	41	thick skin	17,402.61	10,420.15	12,947.05	−0.24705	−0.41192	−0.52184
woman	25	thick skin	21,710.78	15,163.14	10,729.85	−0.16012	−0.36436	−0.51161
woman	21	thick skin	13,763.56	18,821.97	12,284.63	−0.26464	−0.34636	−0.52193
woman	38	thick skin	21,157.52	18,606.53	13,015.94	−0.24231	−0.25535	−0.47844
woman	21	thick skin	20,918.06	25,323.8	12,332.97	−0.2101	−0.30769	−0.45672
woman	22	thick skin	22,649.33	15,808.53	12,677.87	−0.29139	−0.5431	−0.4122
woman	42	thick skin	24,388.37	17,285.89	12,367.27	−0.26726	−0.26577	−0.29671
woman	35	thick skin	15,619.37	16,150.21	9100.597	−0.25005	−0.22118	−0.65626
woman	40	thick skin	25,772.72	13,484.73	14,344.91	−0.25946	−0.26654	−0.48198
woman	20	thick skin	20,317.86	21,325.81	10,002.08	−0.26917	−0.20798	−0.50449
woman	36	thick skin	21,629.53	15,553.38	5893.244	−0.27312	−0.26247	−0.45653
woman	22	thick skin	17,817.65	18,888.61	8938.635	−0.21555	−0.41138	−0.50294
woman	27	thick skin	16,086.12	14,359.22	10,073.25	−0.19032	−0.4437	−0.52105
woman	33	thick skin	29,350.65	14,295.79	10,449.25	−0.19201	−0.3618	−0.61163
woman	43	thick skin	19,725.72	13,739.48	5326.204	−0.27929	−0.56421	−0.58129

**Table 4:** Women aged 20–44 years with thin skin.

Gender	Age	Morfotype	T0	T1 (after 15 Days)	T2 (after 30 Days)	Variation T0–T1	Variation T1–T2	Variation T0–T2
woman	40	thin skin	14,856.64	14,659.79	10,282.07	−0.19769	−0.45025	−0.417752708
woman	39	thin skin	20,203.61	9950.986	6741.714	−0.33279	−0.39844	−0.384509539
woman	29	thin skin	13,531.79	16,246.02	8519.241	−0.2714	−0.29873	−0.45254261
woman	30	thin skin	25,857.01	13,610.23	6701.851	−0.29547	−0.35467	−0.453228038
woman	21	thin skin	12,558.45	23,322.95	10,445.41	−0.31848	−0.48445	−0.559127736
woman	39	thin skin	15,168.59	19,138.73	9126.46	−0.23645	−0.51983	−0.598058721
woman	21	thin skin	19,324.44	18,007.18	13,478.79	−0.23456	−0.5041	−0.682192745
woman	22	thin skin	22,778.28	12,896.46	11,048.87	−0.23009	−0.34021	−0.550126418
woman	34	thin skin	14,976.92	17,604.92	6017.533	−0.25475	−0.33446	−0.464807323
woman	23	thin skin	21,208.37	12,534.36	10,462.61	−0.32922	−0.22281	−0.480560644
woman	43	thin skin	23,557.85	11,633.06	5562.866	−0.24714	−0.2845	−0.281096668
woman	39	thin skin	19,372.53	11,835.07	13,093.66	−0.21267	−0.45446	−0.640864258
woman	40	thin skin	21,858.02	12,505.91	6213.073	−0.26741	−0.40473	−0.406377765
woman	26	thin skin	21,864.14	15,831.18	9467.97	−0.29802	−0.45474	−0.419136573
woman	39	thin skin	20,158.94	15,441.02	5432.492	−0.28213	−0.35764	−0.589632856
woman	39	thin skin	27,340.5	14,528.24	5852.801	−0.37548	−0.35087	−0.402082377
woman	30	thin skin	14,669.51	16,915.49	6809.654	−0.28109	−0.30127	−0.49919779
woman	41	thin skin	27,290.64	17,353.8	11,390.18	−0.22648	−0.36085	−0.429913902

**Table 5:** Women aged 45–79 years with thin skin.

Gender	Age	Morfotype	T0	T1 (after 15 Days)	T2 (after 30 Days)	Variation T0–T1	Variation T1–T2	Variation T0–T2
woman	61	thin skin	17,419.04	13,222.26	12,572.03	−0.22151	−0.26469	−0.53172
woman	78	thin skin	25,416.18	15,464.7	12,474.05	−0.21739	−0.39368	−0.54539
woman	65	thin skin	23,029.81	11,858.1	17,326.69	−0.33438	−0.23176	−0.46488
woman	52	thin skin	21,059.87	7161.12	4685.338	−0.25074	−0.45596	−0.41192
woman	62	thin skin	23,122.47	9442.994	5896.452	−0.30268	−0.39162	−0.51244
woman	63	thin skin	22,111.97	13,937.59	7941.297	−0.24551	−0.43904	−0.49383
woman	73	thin skin	19,581.79	11,306.28	10,303.85	−0.17622	−0.33627	−0.35882
woman	56	thin skin	19,871.3	14,105.56	7650.626	−0.16301	−0.3409	−0.57597
woman	75	thin skin	18,301.19	13,186.9	12,656.31	−0.29245	−0.24376	−0.59068
woman	74	thin skin	30,445.12	5364.362	8965.446	−0.27953	−0.3667	−0.58074
woman	70	thin skin	17,328.6	15,155.7	11,080.6	−0.30591	−0.37437	−0.35348
woman	56	thin skin	25,038.09	16,177.78	15,312.62	−0.28422	−0.22559	−0.5238

**Table 6:** Women aged 45–79 years with thick skin.

Gender	Age	Morfotype	T0	T1 (after 15 Days)	T2 (after 30 Days)	Variation T0–T1	Variation T1–T2	Variation T0–T2
woman	58	thick skin	16,323.93	8479.063	3292.422	−0.25125	−0.49625	−0.60145
woman	57	thick skin	22,108	14,748.82	12,220.67	−0.23177	−0.41986	−0.48853
woman	70	thick skin	26,814.13	16,498.7	5668.684	−0.21419	−0.27426	−0.52881
woman	47	thick skin	13,280.87	14,591.53	12,312.92	−0.20888	−0.33456	−0.49183
woman	71	thick skin	25,069.57	14,874.16	8416.378	−0.28869	−0.23043	−0.55148
woman	47	thick skin	12,788.69	11,151.03	9410.778	−0.23684	−0.53932	−0.49358
woman	73	thick skin	26,946.25	6323.335	12,655.03	−0.26781	−0.42447	−0.25357
woman	65	thick skin	14,542.01	16,447.75	11,554.79	−0.29611	−0.38283	−0.48363
woman	77	thick skin	24,006.04	15,345.53	8652.329	−0.25607	−0.18304	−0.53469
woman	60	thick skin	15,588.92	6529.214	3892.93	−0.20668	−0.36638	−0.47328
woman	59	thick skin	25,407.86	9130.92	8836.921	−0.38194	−0.39855	−0.50193
woman	52	thick skin	21,407.59	20,734.31	9774.793	−0.23089	−0.35803	−0.53056
woman	67	thick skin	20,972.47	11,582.94	6414.549	−0.19481	−0.36935	−0.43018

**Table 7:** Men aged 20–44 years with thin skin.

Gender	Age	Morfotype	T0	T1 (after 15 Days)	T2 (after 30 Days)	Variation T0–T1	Variation T1–T2	Variation T0–T2
man	22	thin skin	15,441.51	8058.827	10,900.35	−0.29414	−0.3433	−0.67155
man	38	thin skin	17,987.74	24,381.65	11,319.21	−0.26407	−0.4042	−0.44536
man	42	thin skin	21,460.09	12,830.37	7291.989	−0.32289	−0.26193	−0.54685
man	25	thin skin	13,629.42	12,524.99	14,333.77	−0.30566	−0.35965	−0.51646
man	33	thin skin	24,439.79	11,829.38	9064.887	−0.1654	−0.33039	−0.55757
man	28	thin skin	19,079.44	12,763.28	8435.904	−0.29401	−0.30406	−0.48223
man	40	thin skin	15,474.12	12,443.79	8812.15	−0.27363	−0.38145	−0.51241
man	20	thin skin	18,426.9	22,739.4	9289.903	−0.32127	−0.42606	−0.55754
man	33	thin skin	23,363.19	8820.136	6971.339	−0.26582	−0.40454	−0.26165
man	20	thin skin	25,469.44	20,303.33	7732.896	−0.263	−0.1389	−0.5145
man	43	thin skin	19,569.31	17,814.12	15,135.32	−0.27692	−0.54808	−0.57172
man	23	thin skin	14,336.16	13044.86	7647.049	−0.28657	−0.46637	−0.7655
man	31	thin skin	25,484.01	11,694.79	7871.778	−0.26107	−0.42648	−0.4733
man	43	thin skin	22,310.81	19,393.61	11,919.84	−0.20431	−0.25407	−0.41324
man	20	thin skin	17,574.37	11,471.49	9462.175	−0.19931	−0.47779	−0.36172
man	39	thin skin	34,783.54	14,312.5	10,801.5	−0.29664	−0.31486	−0.68807
man	43	thin skin	29,298.21	12,983.43	6383.787	−0.32497	−0.303	−0.45169

**Table 8:** Men aged 20–44 years with thick skin.

Gender	Age	Morfotype	T0	T1 (after 15 Days)	T2 (after 30 Days)	Variation T0–T1	Variation T1–T2	Variation T0–T2
man	35	thick skin	13,504.56	11,002.77	8769.011	−0.34453	−0.37039	−0.52224
man	20	thick skin	23,363.95	11,097.35	11,571.52	−0.28439	−0.43513	−0.46543
man	26	thick skin	21,946.42	12,780.37	7106.345	−0.19107	−0.34253	−0.56476
man	42	thick skin	20,500.52	20,006.93	9262.576	−0.20305	−0.22166	−0.45856
man	39	thick skin	23,570.77	9126.694	13,663.99	−0.27608	−0.31445	−0.55604
man	23	thick skin	14,024.85	13,879.73	9438.054	−0.2234	−0.4239	−0.4753
man	36	thick skin	23,031.65	16,554.58	9160.698	−0.1519	−0.39967	−0.72973
man	22	thick skin	14,298.14	14,703.48	5788.73	−0.22151	−0.61316	−0.65426
man	24	thick skin	21,775.41	15,118.72	10,466.18	−0.28638	−0.43111	−0.64857
man	30	thick skin	23,596.99	15,511.99	8382.829	−0.26914	−0.38238	−0.34909
man	41	thick skin	18,421.35	9628.675	7581.488	−0.23621	−0.27209	−0.51917
man	27	thick skin	21,234.31	11,025.03	7668.519	−0.23544	−0.49562	−0.58701
man	29	thick skin	21,388.74	18,525.42	11,180.54	−0.22991	−0.29638	−0.45992
man	39	thick skin	17,299.08	11,087.07	11,982.59	−0.28764	−0.3451	−0.59695
man	34	thick skin	12,300.81	7485.794	10,243.98	−0.21815	−0.20623	−0.61559
man	36	thick skin	20,644.42	19,360.15	8883.416	−0.20029	−0.55902	−0.34216
man	30	thick skin	14,828.94	15,561.17	13,180.15	−0.20932	−0.1191	−0.54405
man	22	thick skin	21,806.73	12,086.23	11,761.94	−0.22309	−0.39517	−0.37899

**Table 9:** Men aged 45–79 years with thin skin.

Gender	Age	Morfotype	T0	T1 (after 15 Days)	T2 (after 30 Days)	Variation T0–T1	Variation T1–T2	Variation T0–T2
man	68	thin skin	22,753.91	15,995.3	6618.532	−0.29174	−0.31335	−0.50401
man	66	thin skin	28,601.41	20,294.36	8022.655	−0.30298	−0.44504	−0.59153
man	72	thin skin	20,106.63	15,694.87	12,203.99	−0.25393	−0.42665	−0.56191
man	64	thin skin	12,395.28	13,064.76	11,823.52	−0.19969	−0.36154	−0.4936
man	66	thin skin	18,792.14	16,220.73	8608.858	−0.24445	−0.24191	−0.49859
man	51	thin skin	19,779.73	13,234.81	11,544.93	−0.27778	−0.47901	−0.47789
man	71	thin skin	21,685.06	16,855.38	9203.279	−0.2476	−0.46561	−0.53462
man	67	thin skin	27,632.08	16,045.52	5740.691	−0.16709	−0.31257	−0.44389
man	45	thin skin	21,421.54	13,362.48	12,745.55	−0.31841	−0.10632	−0.43486
man	61	thin skin	20,463.37	20,438.86	7064.203	−0.27303	−0.48643	−0.5018
man	45	thin skin	22,190.88	12,283.24	6287.826	−0.27109	−0.50093	−0.38977
man	59	thin skin	31,240.46	12,282.47	10,750.97	−0.23528	−0.40928	−0.56381
man	68	thin skin	18,731.3	6105.996	11,601.62	−0.19785	−0.46691	−0.48717
man	57	thin skin	11,530.42	9568.712	9638.318	−0.29417	−0.38979	−0.399
man	77	thin skin	23,881.42	16,980	13,906.85	−0.24766	−0.40576	−0.60858
man	54	thin skin	34,756.3	14,585.75	7975.23	−0.21125	−0.32971	−0.4694
man	48	thin skin	17,181.61	14,004.83	11,923.04	−0.27511	−0.3419	−0.57653

**Table 10:** Men aged 45–79 years with thick skin.

Gender	Age	Morfotype	T0	T1 (after 15 Days)	T2 (after 30 Days)	Variation T0–T1	Variation T1–T2	Variation T0–T2
man	60	thick skin	18,775.54	13,640.96	9129.175	−0.26143	−0.31108	−0.4349
man	74	thick skin	15,266.35	13,420.45	10,128.88	−0.2511	−0.382	−0.48643
man	70	thick skin	32,121.07	10,729.92	10,612.15	−0.23929	−0.20298	−0.57092
man	57	thick skin	13,109.74	7501.458	10,636.84	−0.26788	−0.27729	−0.53054
man	70	thick skin	17,561.48	14,173.26	9695.481	−0.20671	−0.45216	−0.53538
man	62	thick skin	20,190.63	10,756.54	5990.925	−0.19367	−0.38422	−0.53004
man	77	thick skin	25,366.16	4971.146	11,953.99	−0.23927	−0.3434	−0.52569
man	56	thick skin	15,714.86	16,694.39	5168.417	−0.18511	−0.38654	−0.79778
man	72	thick skin	16,077.08	22,204.14	14,848.51	−0.24696	−0.4057	−0.57643
man	49	thick skin	25,849.22	15,162.52	9313.583	−0.34209	−0.48004	−0.41725
man	72	thick skin	17,947.48	8807.184	11,369.65	−0.22871	−0.1383	−0.40835
man	61	thick skin	17,232.19	15,218.84	9939.49	−0.22296	−0.40836	−0.47662
man	72	thick skin	18,160.72	15,743.71	7314.114	−0.26902	−0.27921	−0.45876
man	52	thick skin	21,900.47	8874.207	9879.819	−0.35481	−0.51322	−0.48008

## Data Availability

Data are available upon reasonable request.
